# The association between residential segregation and stillbirths in Brazil—a cross-sectional study

**DOI:** 10.1186/s12884-025-08329-x

**Published:** 2025-11-04

**Authors:** Mio Kushibuchi, José Firmino Sousa Filho, Andrêa J.F. Ferreira, Hannah Blencowe, Gervásio Ferreira Santos, Mauricio L. Barreto, Enny S. Paixão

**Affiliations:** 1 Centro de Integração de Dados e Conhecimentos para Saúde (CIDACS), Centro de Integração de Dados e Conhecimentos de Saúde Edf Tecnocentro, Sl 315, R. Mundo, 121, Trobogy, Salvador, Bahia Brazil; 2 London School of Hygiene & Tropical Medicine (LSHTM), Keppel Street, London, UK

**Keywords:** Stillbirth, Income segregation, Racial segregation, Inequities in health, Latin America

## Abstract

**Background:**

Segregation is the degree to which two or more groups live separately. While US research has linked segregation to increased stillbirth risk, studies from Latin America have yet to explore this. This study investigated the association between the racial and income segregation index (SI) and stillbirth prevalence in Brazil.

**Methods:**

We used nationwide birth data from Brazil in 2018 (live births from Live Birth Information System, SINASC, and stillbirths from Mortality Information System, SIM). Income and racial SI were calculated using the 2010 national census and analyzed as quintiles with the least segregated group as the reference. Odds ratios were calculated using a logistic regression model, adjusting for infant sex, maternal age, education, previous fetal loss, and the municipal level percentage of the population earning less than half the minimum wage. The sub-analysis was stratified by city size, area-level stillbirth prevalence, and stillbirth type (intrapartum or antepartum).

**Results:**

Two million seven hundred seventy-one thousand two hundred seventy-two live-born and stillborn in 2018 were included in the analysis. Women in municipalities with high income and racial SI were older, had more education, and had more previous fetal loss. Women in municipalities with the highest income SI had a 25.1% higher risk of delivering a stillbirth (95% CI: 1.202–1.303). Those in the highest racial SI municipalities had a 5.5% lower risk of delivering a stillbirth compared to those in the quintile with the lowest racial SI (95% CI: 0.908–0.984). In regions with low stillbirth prevalence, a dose-response relationship was observed between income segregation and stillbirth, with the risk of stillbirth among those with the highest income SI being more than twice that of the least segregated (OR 2.086, 95% CI: 1.494–2.911). In larger cities, racial and income segregation were associated with reduced odds of stillbirth. The effect of income SI was larger for intrapartum stillbirths.

**Conclusions:**

We observed that income segregation increases the odds of stillbirth, especially in municipalities with low stillbirth prevalence, while the association for racial segregation was less consistent.

**Supplementary Information:**

The online version contains supplementary material available at 10.1186/s12884-025-08329-x.

## Background

In 2021, an estimated 1.9 million late gestation (≥ 28 weeks) stillbirths occurred worldwide, according to the World Health Organization (WHO) [1–4]. Notably, 98% of these stillbirths took place in low or middle-income countries (LMIC) [5]. Stillbirth disproportionally affects disadvantaged populations, with social markers such as low income, ethnic minority status, occupation, low social class, and low education being associated with increased stillbirth risk [6–9].

Brazil is classified as an upper-middle-income country, with one of the highest inequalities globally [10]. Marked socioeconomic disparities exist between individuals, with the urban population in extreme poverty residing in precarious conditions such as favelas, while the wealthier white population also self-segregate [11]. Regional disparity is also notable, with the North and Northeastern regions, which have a more Afro-descendent population, being less affluent than the southern regions. These socioeconomic and spatial divides translate into unequal access to healthcare and are reflected in perinatal outcomes. Brazil has a universal health coverage system, the Unified Health System (SUS), which provides high overall coverage of medicalized deliveries in Brazil. In 2021, the national stillbirth rate was 6.98 per 1,000 births [12], but remarkable regional disparity exists, with the odds in the Northeast region being twice as high as in the other regions [13]. These disparities are partly due to the unequal distribution of healthcare facilities, compounded by variations in the quality of care across different facility types [13].

The inequality of Brazilian society can also be seen in the residential segregation. Residential segregation is defined as the degree to which two or more social groups live separately from one another in urban environments [14, 15]. It represents the spatial inequity in resource allocation, a social determinant of health [16]. In Brazil, this is a consequence of both the long-standing racism towards the Afro-descendants and the indigenous people rooted in the colonial history, and the unplanned urbanization since the 1950 s [11, 17]. Structural racism, manifested as residential area segregation, denies Afro-descendants and Indigenous people in urban areas their access to housing, education, stable employment, and quality healthcare, and keeps them marginalized in precarious neighborhoods [11, 18]. The condition is similar to that in many Latin American cities [11, 19], but both the history and the circumstances differ from those of the US, where most studies on segregation have been conducted [11, 18].

Health outcomes shown to be linked to racial segregation in the US include overall mortality, premature death, cancer, heart disease, infant mortality, low birth weight, preterm birth, and infectious diseases like gonorrhea and tuberculosis [20]. Studies from the US have not used the income segregation. From Brazil, studies have reported associations between high income segregation and elevated risk of homicides [19], COVID-19 mortality [21], type 2 diabetes mellitus, hypertension, breast cancer [21, 22], TB treatment outcome [23], and BMI change [18]. Racial segregation was only investigated in studies of TB treatment and BMI change, where the effect was similar to that of the income SI [18, 23].

Stillbirth is also evidenced to be associated with segregation from studies from the US. Racial segregation is shown to increase the risk of stillbirth by 15% among Black mothers in segregated areas but not among White mothers [24]. Another study showed that reducing segregation could potentially lead to an 8–15% reduction in stillbirths among Black mothers living in segregated areas [25]. Although the impact of income segregation on stillbirth has not been specifically investigated, it is estimated that income segregation increases the risk of neonatal mortality and severe morbidity by 40% in New York City [26]. There is currently no evidence from Brazil examining the association between income and racial segregation and stillbirths.

Therefore, this study aimed to investigate the association between income and racial segregation and stillbirth in Brazil.

## Materials and methods

### Study design

This is a nationwide cross-sectional study of all individuals born to mothers who gave birth between January 1 and December 31, 2018, in Brazil. Data was obtained from the Brazilian government’s open-access administrative data, DATASUS (Departamento de Informações do Sistema Único de Saúde).

### Data source

Stillbirth data were obtained from the Brazilian Mortality Information System (SIM: Sistema de Informações de mortalidade). Live birth data were obtained from the Live Birth Information System (SINASC: Sistema de Informações de Nascidos Vivos). The SINASC and SIM data were combined to create a unique dataset including all births (live and still) in Brazil. This dataset includes information on maternal education, maternal age, type of gestation, previous fetal loss, municipality of residence, and the sex of the newborn for both live births and stillbirths. For stillbirths, the type of stillbirth (antepartum or intrapartum) is also recorded. Individuals with a maternal age of 14 years or younger or older than 49 years were excluded from this study, as the WHO defines the childbearing age as 15 to 49 years, and limited information is available on the outcomes of pregnancies outside this age range [27]. Those without a municipality code were also excluded from the analyses.

Municipality-level information was obtained from the 2010 census, the most recent available version, conducted by the IBGE (Instituto Brasileiro de Geografia e Estatística). These included the data necessary to calculate the income and racial segregation index, as well as other municipality-level variables such as the percentage of people earning below half of the minimum wage and population density. Brazil has 5,569 municipalities, with the largest in population being approximately 11.4 million, and the largest in area being nearly 160,000 square kilometers [28]. The minimum wage was 510 Brazilian Reais (equivalent to 290 US Dollars in 2010) when the census was conducted. Race is self-declared, from black (preta), brown (parda), white (branca), indigenous (indigena), and Asian (amarela). Parda is an official racial/ethnic category used in the census, encompassing individuals who self-identify with mixed or intermediate skin tone.

### Exposure

Segregation index (SI) is an index that measures the evenness dimension of segregation, which is one of the five dimensions of segregation described by Massey and Denton (evenness, exposure, concentration, centralization, and clustering) [14]. The income and racial SI for Brazilian municipalities were calculated by Sousa et al.; the methodology and distribution are described in detail by them [29]. In short, SI was calculated for each municipality using the Dissimilarity index formula proposed by Duncan and widely used in studies of segregation [15]. It measures “how evenly across the municipalities are the minorities distributed”. It ranges from 0 (complete integration) to 1 (complete segregation), representing the proportion of families that would need to relocate for an even distribution across census tracts. For income segregation, we used the two-minimum-wage cut-off, as recommended by the authors [11]. Although no particular recommendation for racial SI is given due to the heterogeneity of the Brazilian racial distribution, we used the black versus white index in our study to ensure comparability with the US literature.

Each individual was assigned the segregation index corresponding to their municipality of birth. The racial SI was calculated for Black individuals compared to white individuals, but the municipal-level value was then assigned to all individuals born in that municipality, regardless of their race. The exposure was then categorized into quintiles based on the distribution across individuals (rather than municipalities), as done in previous studies, with the first quintile being the least segregated [23].

### Outcome

The outcome, stillbirth, is defined as the death of a fetus weighing 500 g or more, or older than 22 weeks of gestational age [30]. Stillborn individuals registered in the SIM were compared to the live-born individuals registered in SINASC. We also conducted separate analyses by stillbirth type, antepartum or intrapartum, which is a required field in the SIM registry. Intrapartum stillbirths are stillbirths that happen after initiation of labor and before delivery, and antepartum stillbirths are any stillbirth before that; previous studies report that most intrapartum stillbirths can be prevented with adequate monitoring and obstetric intervention [9].

### Covariates

A logical model was proposed to explain the relationship between income and racial SI and stillbirth (Supplementary Fig. 1) based on previous studies [9, 31] and apriori hypothesis. Factors we hypothesized to be confounders and are obtainable from our dataset were: For individual SES, maternal age, categorized into 15–19 years, 20–35 years, and 36–49 years; and maternal education, categorized into zero to 3 years, 4 to 7 years, 8 to 11 years, and 12 years or more were included. For area-level SES, the percentages earning half the minimum wage were included as a continuous variable. Previous stillbirth and infant sex were not confounders, but were included as prognostic covariates. Previous stillbirth is self-reported by the mother in both SINASC and SIM, and does not include miscarriages. Unmeasured confounders that we could not obtain in our dataset were family income and the urbanicity of the residence. Additionally, previous studies suggest that race may be an effect modifier; however, we did not have information on maternal race for the SIM [25, 32].

### Statistical analysis

In basic demographics, continuous variables are presented as the median and interquartile range (IQR), and categorical variables are presented as the number and percentage for the overall population and by SI quintiles.

The logistic regression was adjusted for the covariates listed above (maternal age, maternal education, child sex, previous stillbirth, and municipality-level percentage of individuals earning less than half the minimum wage), and the odds ratio (OR) and 95% confidence intervals (95% CI) were calculated.

Three stratified analyses were conducted. The first analysis stratifies by municipality population size, with a cutoff of 500,000 people, under the hypothesis that the association between segregation and stillbirth may differ by city size. The second analysis stratified by municipality-level stillbirth prevalence. For this analysis, we calculated the prevalence of stillbirths for each municipality by dividing the number of stillbirths by the total number of births. This proportion was imputed for each individual as an area-level variable; subsequently, the individuals were divided into quintiles based on the proportion. This is based on a previous study in the US that showed how segregation is only a risk factor for adverse birth outcomes in areas with low stillbirth prevalence [33]. The third sub-analysis was conducted separately for intrapartum and antepartum stillbirths, as previous studies have shown that the risk factors for these two types of stillbirths may differ [34–36].

We performed three post-hoc sensitivity analyses. The first analysis excluded twins and those missing the plurality variable, under the possibility that, because non-singleton pregnancies have a markedly elevated risk of adverse pregnancy-related outcomes, the effect of stillbirth may differ for the non-singletons. The second sensitivity analysis involved stratifying the population by region. Because the racial composition and economic conditions differ greatly by region, the effect of segregation may also vary by region [11]. The last sensitivity analysis involved excluding the area-level percentage of individuals earning below half the minimum wage from the Logistic model. This analysis was performed because this variable may capture similar aspects of socioeconomic structure already reflected in the segregation index, and we wanted to assess whether its inclusion was leading to over-adjustment of the economic context. This analysis was also stratified by tertiles of the percentage of people earning less than half the minimum wage, to investigate effect modifications by area-level poverty.

All analyses were done using STATA SE 18.0 and R version 4.4.1.

## Results

**Fig. 1:**
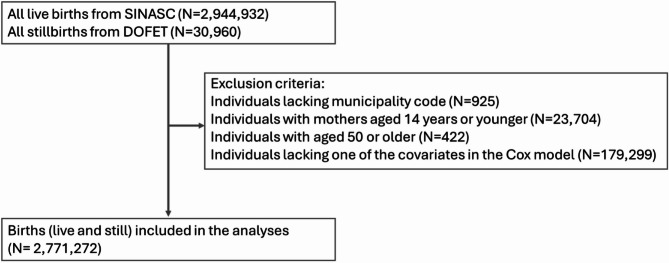
The participant flowchart

Our cohort included 2,771,272 individuals born in 2018, after excluding 23,704 births from mothers aged younger than 15 years and 422 births from mothers older than 49 years (Fig. 1). The primary demographics for the overall population and quintiles of SI are shown in Table 1. The percentages of older mothers with low and high education were higher in high-income SI quintiles. The prevalence of prior fetal loss was 19.4% overall and was higher in the high-income SI quintile (21.3%). For the racial SI quintiles, the same tendency was observed for maternal age and education, but not for prior fetal loss. Regarding the municipality-level variables, the first to third quintiles of income SI consisted primarily of participants from the South and Southeast regions, whereas more than half of the participants in the highest income SI quintile were from the Northeast region. The percentage of individuals earning below half the minimum wage was higher in the fourth income quintile (42%). For racial-SI quintiles, the regional distribution was more even compared to that of income SI, but there were more Southeastern regions in the low racial-SI quintiles and more Southern regions in the high racial-SI quintiles. For both SI quintiles, the median population of municipalities was higher in those with high SI quintiles. Details of the municipalities included in each quintile are described in Supplementary Table 1. The demographics by the outcome status are described in Supplementary Table 2; stillborn babies were more likely to be males and twins, and the mothers with stillbirths were older, less educated, more likely to be nulliparous, and had a higher proportion of previous fetal loss.

**Table 1:** Basic demographics of the study population by quintiles of segregation index

	Overall	Income SI quintiles					Racial SI quintles				
		1st	2nd	3rd	4th	5th	1st	2nd	3rd	4th	5th
Number of participants	2,771,272	554,255	554,255	554,254	554,254	554,254	554,255	554,255	554,254	554,254	554,254
Sex											
Male	1,419,570 (51.2)	283,602 (51.2)	284,260 (51.3)	284,202 (51.3)	283,590 (51.2)	283,916 (51.2)	283,754 (51.2)	283,582 (51.2)	283,590 (51.2)	283,947 (51.2)	284,697 (51.4)
Female	1,351,702 (48.8)	270,653 (48.8)	269,995 (48.7)	270,052 (48.7)	270,664 (48.8)	270,338 (48.8)	270,501 (48.8)	270,673 (48.8)	270,664 (48.8)	270,307 (48.8)	269,557 (48.6)
Maternal age											
Median [IQR]	27.08 (6.67)	26.89 (6.54)	27.02 (6.59)	27.10 (6.65)	26.95 (6.75)	27.45 (6.82)	26.50 (6.57)	26.98 (6.60)	27.26 (6.63)	27.50 (6.71)	27.17 (6.80)
15 to 19 years	406,010 (14.7)	80,547 (14.5)	79,926 (14.4)	80,395 (14.5)	86,879 (15.7)	78,263 (14.1)	90,235 (16.3)	81,131 (14.6)	77,038 (13.9)	73,804 (13.3)	83,802 (15.1)
20 to 34 years	1,926,156 (69.5)	392,592 (70.8)	390,171 (70.4)	386,449 (69.7)	379,891 (68.5)	377,053 (68.0)	388,597 (70.1)	389,304 (70.2)	387,668 (69.9)	383,630 (69.2)	376,957 (68.0)
35 to 49 years	439,106 (15.8)	81,116 (14.6)	84,158 (15.2)	87,410 (15.8)	87,484 (15.8)	98,938 (17.9)	75,423 (13.6)	83,820 (15.1)	89,548 (16.2)	96,820 (17.5)	93,495 (16.9)
Maternal education											
none to 3 years	62,640 (2.3)	10,209 (1.8)	11,038 (2.0)	12,025 (2.2)	15,378 (2.8)	13,990 (2.5)	12,729 (2.3)	10,789 (1.9)	10,815 (2.0)	11,830 (2.1)	16,477 (3.0)
4 to 7 years	410,100 (14.8)	79,629 (14.4)	77,350 (14.0)	79,848 (14.4)	87,079 (15.7)	86,194 (15.6)	88,406 (16.0)	78,631 (14.2)	76,779 (13.9)	73,792 (13.3)	92,492 (16.7)
8 to 11 years	1,710,744 (61.7)	364,612 (65.8)	349,991 (63.1)	338,775 (61.1)	337,293 (60.9)	320,073 (57.7)	365,273 (65.9)	349,887 (63.1)	333,386 (60.2)	335,299 (60.5)	326,899 (59.0)
12 or more years	587,788 (21.2)	99,805 (18.0)	115,876 (20.9)	123,606 (22.3)	114,504 (20.7)	133,997 (24.2)	87,847 (15.8)	114,948 (20.7)	133,274 (24.0)	133,333 (24.1)	118,386 (21.4)
Gestation type											
Singleton	2,708,059 (97.7)	541,806 (97.8)	541,760 (97.7)	541,539 (97.7)	541,817 (97.8)	541,137 (97.6)	542,305 (97.8)	541,958 (97.8)	541,473 (97.7)	540,827 (97.6)	541,496 (97.7)
Twin or more	61,424 (2.2)	12,170 (2.2)	12,139 (2.2)	12,374 (2.2)	12,103 (2.2)	12,638 (2.3)	11,549 (2.1)	11,885 (2.1)	12,480 (2.3)	13,160 (2.4)	12,350 (2.2)
missing	1789 (0.1)	279 (0.1)	356 (0.1)	341 (0.1)	334 (0.1)	479 (0.1)	401 (0.1)	412 (0.1)	301 (0.1)	267 (0.0)	408 (0.1)
Gestation weeks											
< 22 weeks	2790 (0.1)	500 (0.1)	534 (0.1)	528 (0.1)	497 (0.1)	731 (0.1)	580 (0.1)	570 (0.1)	516 (0.1)	542 (0.1)	582 (0.1)
22 to 27 weeks	18,628 (0.7)	3537 (0.6)	3521 (0.6)	3834 (0.7)	3489 (0.6)	4247 (0.8)	3749 (0.7)	3755 (0.7)	3635 (0.7)	3836 (0.7)	3653 (0.7)
28 to 31 weeks	31,292 (1.1)	6077 (1.1)	6030 (1.1)	6303 (1.1)	6195 (1.1)	6687 (1.2)	6306 (1.1)	6210 (1.1)	6277 (1.1)	6231 (1.1)	6268 (1.1)
32 to 36 weeks	265,091 (9.6)	52,561 (9.5)	52,930 (9.5)	54,076 (9.8)	52,032 (9.4)	53,492 (9.7)	52,130 (9.4)	53,475 (9.6)	52,939 (9.6)	52,835 (9.5)	53,712 (9.7)
37 to 41 weeks	2,352,641 (84.9)	473,724 (85.5)	470,668 (84.9)	469,813 (84.8)	469,824 (84.8)	468,612 (84.5)	468,158 (84.5)	471,392 (85.0)	471,907 (85.1)	473,727 (85.5)	467,457 (84.3)
42 or more weeks	69,098 (2.5)	12,291 (2.2)	13,104 (2.4)	14,079 (2.5)	15,125 (2.7)	14,499 (2.6)	14,619 (2.6)	13,294 (2.4)	13,087 (2.4)	12,045 (2.2)	16,053 (2.9)
missing	31,732 (1.1)	5565 (1.0)	7468 (1.3)	5621 (1.0)	7092 (1.3)	5986 (1.1)	8713 (1.6)	5559 (1.0)	5893 (1.1)	5038 (0.9)	6529 (1.2)
Parity											
Nulliparous	1,735,971 (62.6)	350,776 (63.3)	347,998 (62.8)	346,043 (62.4)	348,622 (62.9)	342,532 (61.8)	355,443 (64.1)	347,856 (62.8)	345,990 (62.4)	343,786 (62.0)	342,896 (61.9)
Multiparous	1,027,396 (37.1)	202,785 (36.6)	205,460 (37.1)	206,823 (37.3)	204,094 (36.8)	208,234 (37.6)	197,871 (35.7)	205,110 (37.0)	206,878 (37.3)	208,289 (37.6)	209,248 (37.8)
missing	7905 (0.3)	694 (0.1)	797 (0.1)	1388 (0.3)	1538 (0.3)	3488 (0.6)	941 (0.2)	1289 (0.2)	1386 (0.3)	2179 (0.4)	2110 (0.4)
Previous stillbirth											
No history	2,234,364 (80.6)	451,507 (81.5)	450,672 (81.3)	449,521 (81.1)	446,347 (80.5)	436,317 (78.7)	448,053 (80.8)	447,975 (80.8)	447,297 (80.7)	444,199 (80.1)	446,840 (80.6)
Stillbirth history	536,908 (19.4)	102,748 (18.5)	103,583 (18.7)	104,733 (18.9)	107,907 (19.5)	117,937 (21.3)	106,202 (19.2)	106,280 (19.2)	106,957 (19.3)	110,055 (19.9)	107,414 (19.4)
Municipality-level variables											
Region											
Central west	240,141 (8.7)	64,773 (11.7)	53,246 (9.6)	65,633 (11.8)	12,615 (2.3)	43,874 (7.9)	63,988 (11.5)	48,790 (8.8)	107,711 (19.4)	9007 (1.6)	10,645 (1.9)
Northeast	725,320 (26.2)	51,155 (9.2)	74,052 (13.4)	110,420 (19.9)	209,202 (37.7)	280,491 (50.6)	121,863 (22.0)	154,139 (27.8)	152,816 (27.6)	141,478 (25.5)	155,024 (28.0)
North	284,959 (10.3)	41,605 (7.5)	69,597 (12.6)	49,644 (9.0)	88,245 (15.9)	35,868 (6.5)	77,177 (13.9)	58,871 (10.6)	51,976 (9.4)	40,075 (7.2)	56,860 (10.3)
Southeast	1,128,653 (40.7)	256,302 (46.2)	264,235 (47.7)	220,247 (39.7)	194,060 (35.0)	193,809 (35.0)	261,305 (47.1)	218,814 (39.5)	157,931 (28.5)	282,179 (50.9)	208,424 (37.6)
South	392,199 (14.2)	140,420 (25.3)	93,125 (16.8)	108,310 (19.5)	50,132 (9.0)	212 (0.0)	29,922 (5.4)	73,641 (13.3)	83,820 (15.1)	81,515 (14.7)	123,301 (22.2)
Percent with low income*	34.72 (20.18)	30.45 (17.01)	30.40 (18.35)	33.55 (20.71)	41.92 (21.69)	37.29 (20.37)	38.35 (16.63)	33.61 (17.90)	31.52 (19.03)	31.87 (20.04)	38.28 (25.11)
Municipality area	2525 (8110)	1140 (3752)	3547 (12805)	2363 (6373)	3100 (5457)	2473 (8778)	3125 (12001)	2032 (5330)	2471 (4804)	2378 (7795)	2617 (8479)
Municipality population (thousand)	1201.6 (2786.2)	118.2 (174.9)	179.0 (216.8)	324.5 (441.4)	2356.6 (4211.6)	3029.7 (3625.0)	191.3 (235.0)	296.2 (370.7)	571.7 (786.4)	3651.1 (4997.3)	1297.7 (218.7)

*Abbreviations: IQR* interquartile range, *SI* Segregation Index

* Percent with low income was calculated for each municipality as the percent of population earning less than half a minimum wage, divided by the overall population

**Fig. 2:**
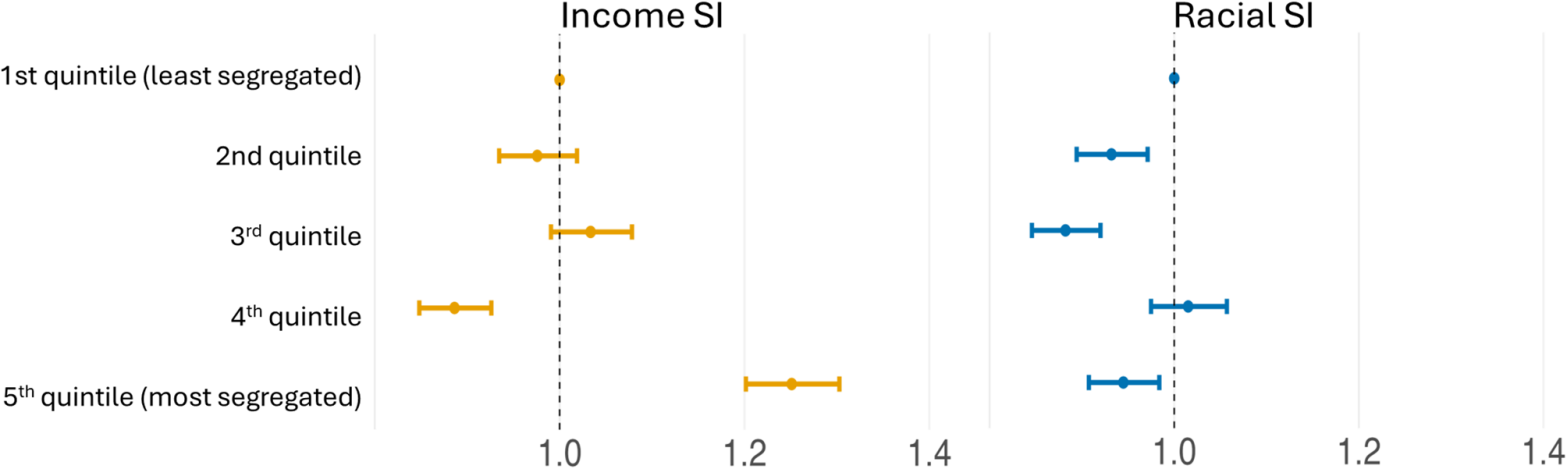
The OR for income and race SI quintiles for the overall analyses. All the OR are adjusted for maternal age, maternal education, child sex, previous stillbirth, and municipality-level percentage earning less than half minimum wage. Abbreviations: OR, odds ratio; SI, segregation index

After adjusting for confounders, women residing in municipalities with the highest income segregation had a 25.1% higher chance of delivering a stillbirth [95% CI: 1.202–1.303] compared to those in municipalities with the lowest income segregation (Fig. 2, Supplementary Table 3). There was no evident effect for the second and third quintiles, and it seemed to be inverted for the fourth quintile. Women residing in municipalities with the highest racial SI had a 5.5% lower chance of delivering a stillbirth [95% CI: 0.908–0.984] compared to those in the quintile with the lowest racial SI. The effect was similar for the third quintile.

**Fig. 3:**
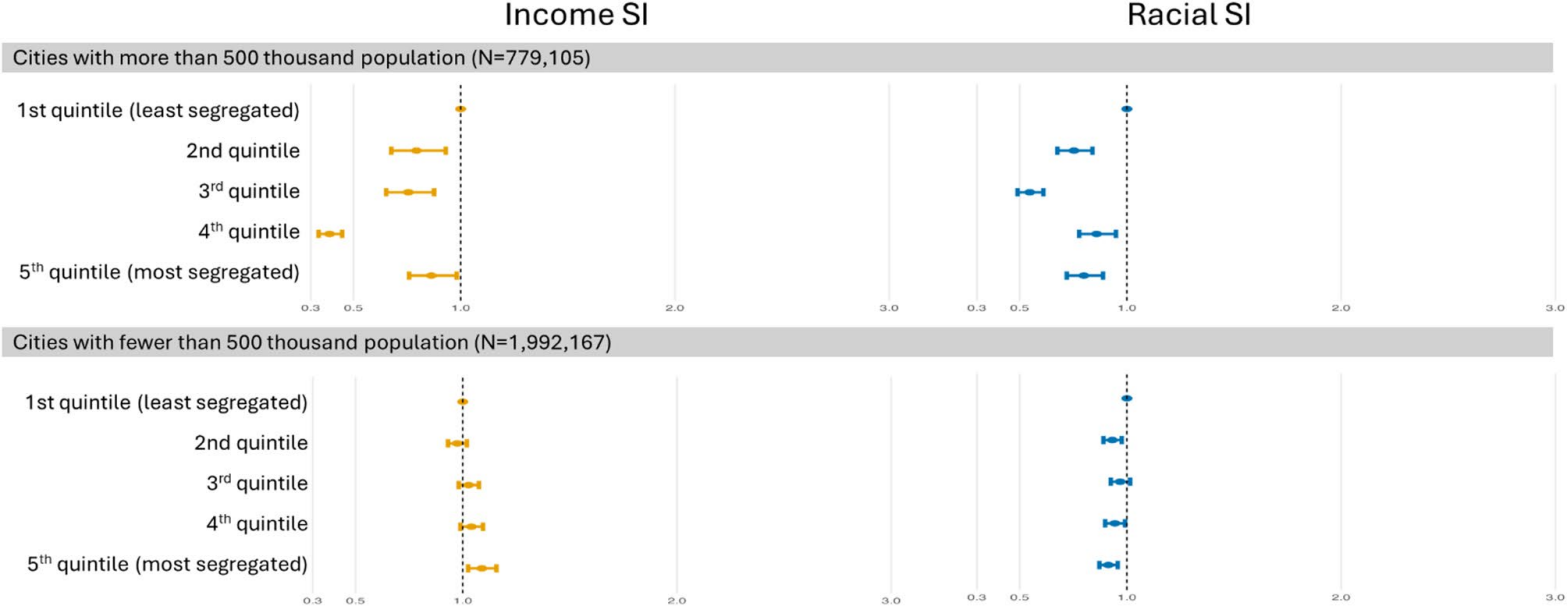
The OR of income and racial SI quintiles, stratified by municipality population size. All the OR are adjusted for maternal age, maternal education, child sex, previous stillbirth, and municipality-level percentage earning less than half minimum wage. Abbreviations: OR, odds ratio; SI, segregation index

Among larger municipalities, both high income SI and racial SI was associated with lower odds of stillbirth compared to living in the least segregated municipalities, with the risk of stillbirth being 14% smaller for the highest income SI quintile and 20% smaller for the highest racial SI quintile, each compared to their lowest SI quintile (95% CI: 0.759–0.982 and 0.719–0.889, respectively)(Fig. 3, Supplementary Table 3). In smaller municipalities, the effect was the same as that of the overall analyses.

**Fig. 4:**
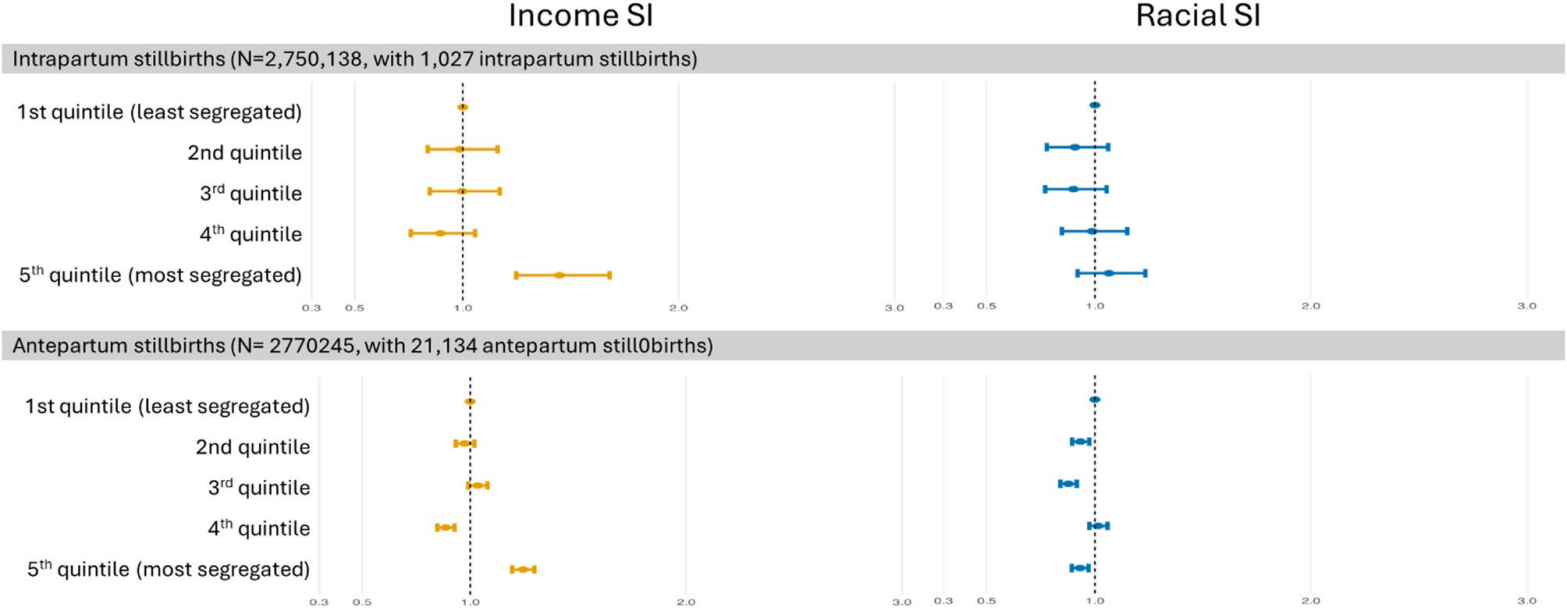
The OR of income and racial SI quintiles for antepartum and intrapartum stillbirth. All the OR are adjusted for maternal age, maternal education, child sex, previous stillbirth, and municipality-level percentage earning less than half minimum wage. Abbreviations: OR, odds ratio; SI, segregation index

In the analyses conducted for only intrapartum stillbirths, the effect of income SI was larger than in the overall analyses (OR: 1.448, 95% CI: 1.248–1.680), whereas the effect for racial SI diminished. For antepartum stillbirths, a similar effect to the overall analyses was observed for both income SI and racial SI (Fig. 4, Supplementary Table 3).

**Fig. 5:**
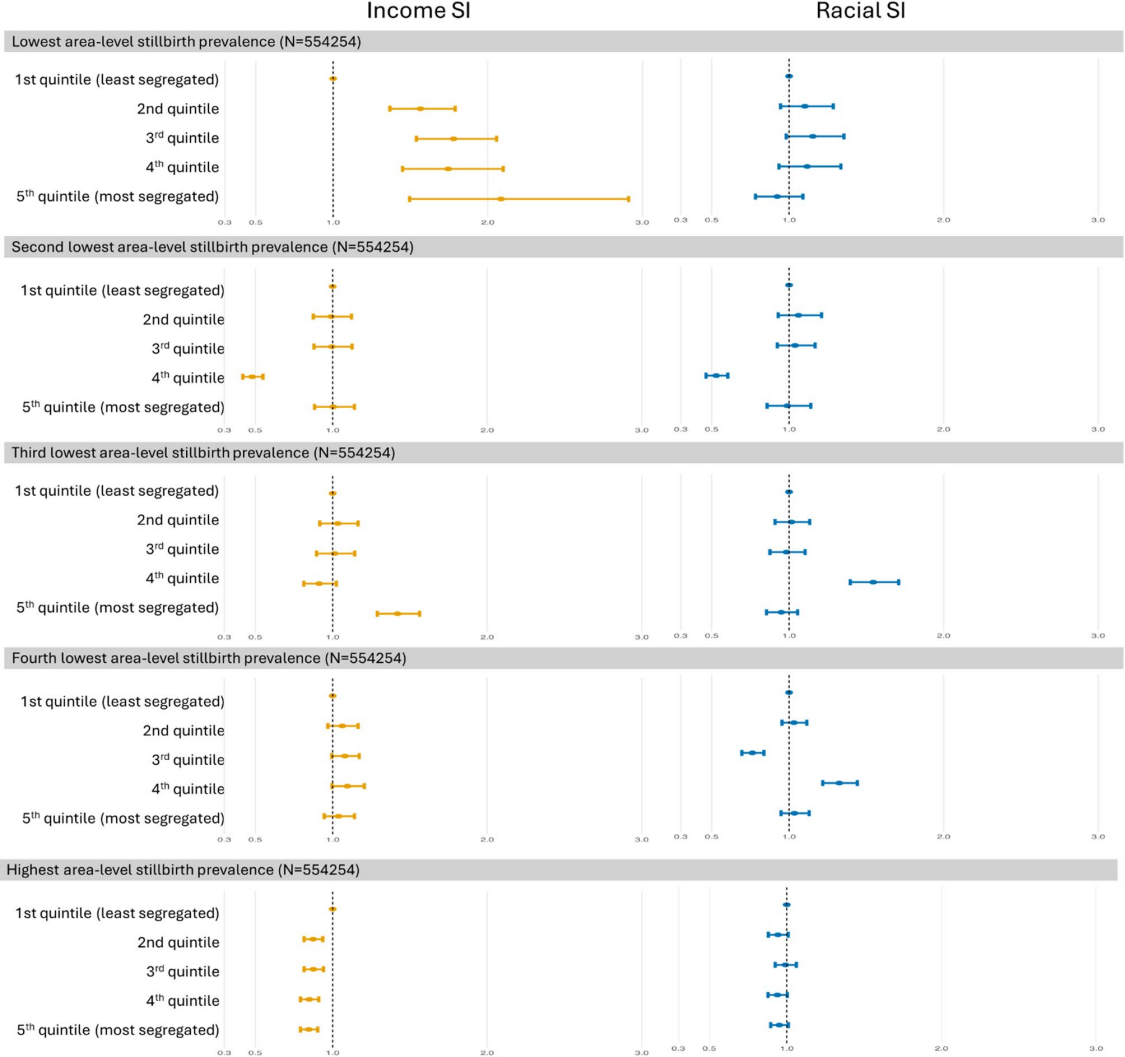
The OR of income and racial SI quintiles stratified by municipality stillbirth prevalence quintiles. All the OR are adjusted for maternal age, maternal education, child sex, previous stillbirth, and municipality-level percentage earning less than half minimum wage. Abbreviations: OR, odds ratio; SI, segregation index

In the analyses stratified by the area-level stillbirth prevalence, high-income SI doubled the risk of stillbirths in areas with low stillbirth prevalence (OR: 2.086, 95% CI: 1.494–2.911) (Fig. 5, Supplementary Table 4). In this area, income SI had a dose-effect relationship with stillbirth odds, with the odds increasing gradually from the second to the fifth quintile. Racial SI had no statistically significant effect. In areas with high stillbirth prevalence, income SI showed an inverted effect on stillbirth, with the risk in the fifth quintile being 15% lower than the least segregated quintile (95%CI: 0.792–0.904). Stillbirth prevalence in each stratum is shown in Supplementary Tables 5, and the municipality-level description of each stratum is in Supplementary Table 6. In short, the areas with the lowest prevalence were predominantly in the South and Southeast regions, characterized by a low percentage of the low-income population. They also had a smaller population (6.5 million on average). The highest prevalence areas were predominantly in the North and Northeast regions, with a high percentage of the low-income population.

The sensitivity analyses excluding twins produced similar results to the overall analyses (Supplementary Fig. 2 and Supplementary Table 7). In the sensitivity analyses stratified by region, the effect was similar in most regions, but with some exceptions. For income SI, its harmful effect was observed in the Southeast and Northeast regions, but not in the other regions. For racial SI, the association with lower odds of stillbirth was observed in the Northeast and Southeast regions. Contrary to the main findings, racial SI appeared to increase the odds of stillbirth in the North region (Supplementary Fig. 3 and Supplementary Table 7).

In the third sensitivity analysis, excluding the area-level poverty variable from the logistic model yielded similar results (Supplementary Fig. 4 and Supplementary Table 7). When we stratify by the percentage of low-income individuals in the area, the effect of income segregation is markedly higher in areas with fewer people living on low incomes. In the areas with few to moderate percentages of low-income people, the higher racial SI seemed to elevate the odds of stillbirth.

## Discussion

This is the first study to investigate the association between income and racial segregation on birth outcomes in any Latin American country. We found that women living in municipalities with higher income segregation had a 25.1% increased likelihood of stillbirth. The effect was particularly pronounced for women living in areas with lower stillbirth prevalence, in smaller cities, and for intrapartum stillbirth. The impact of racial segregation was mixed. While for the overall analyses, higher racial SI was associated with lower odds of stillbirth, the effect was inverted for areas with low stillbirth prevalence and for intrapartum stillbirth.

The association between income segregation and stillbirth, after adjusting for area-level poverty, suggests that the income segregation captures a dimension of socioeconomic context that extends beyond material deprivation alone. While poverty measures the overall level of resource scarcity, segregation captures how that scarcity is distributed and concentrated across space. This effect is especially conspicuous in how the impact of income SI on stillbirth was more substantial in areas with lower stillbirth prevalence, areas with a lower percentage of low-income people, and in the Southeast region. We speculate that this is because in these places, the richer majority can access quality healthcare while the minoritized group is denied access to these resources; however, we could not ascertain this with a stratified analysis for the minoritized group. In areas with high stillbirth prevalence, the majority population also lacks access to quality care; thus, the effect of segregation diminishes. This result aligns with a previous study in the US with low birth weight (LBW) and preterm birth (PTB) [33]. Regarding the low OR observed for the fourth income SI quintile in some stratified analyses, this group included only cities from the South and the Southeast regions, which we assume is the reason for the unnaturally low odds (data not shown).

Income segregation can be linked to stillbirth in several pathways. First, healthcare access may be worse in highly segregated areas. Inadequate antenatal care access leads to worse control of pregnancy complications such as gestational diabetes and hypertension, leading to increased stillbirths [7]. This pathway is also suggested by the strong effect of income SI on intrapartum stillbirths—stillbirths that can, at large, be prevented with adequate monitoring and timely obstetric intervention. Secondly, tobacco, alcohol, and illegal drug use, which are known risk factors of stillbirth, are known to be more prevalent among young adults from highly segregated areas [7, 37]. Third, in Brazil, more segregated areas are often characterized by higher inequality and crime rates, including homicides [19]. This can increase maternal psychological stress, leading to elevated levels of cortisol and blood pressure, causing a compromised immune system and gestational hypertension. Fourth, maternal health may be adversely affected in highly segregated areas due to greater exposure to air pollution and reduced access to sanitation, potentially contributing to stillbirth [8].

Regarding the association between high racial segregation and lower risk of stillbirth, our finding contradicts our initial research hypotheses and should be interpreted cautiously. The apparent association between high racial SI and lower stillbirth can be explained by how cities with high racial SI are those with a lower percentage of black population, which are the wealthier cities concentrated in the Southern and Southeastern regions [11]. These regions typically offer better infrastructure and healthcare access in general, thus leading to the apparent association between high racial SI and lower stillbirth. Our sensitivity analyses of the North region, where we observed the hazardous effects of racial SI, corroborate this, for the North region has a higher percentage of the Afro-descendants and the Indigenous population. Future studies with more information about the individual race and income, as well as the healthcare access, are needed to investigate how racial segregation impacts stillbirth. However, we should note that racial segregation in Brazil is qualitatively different from that of the US, where sharp racial boundaries emerged as a legacy of formal racial segregation. In Brazil, *miscigenação* (racial mixing) has historically shaped society, and racial categories are defined along a continuum of skin color. A large share of the population self-identifies as Pardo (Brown or mixed race), which is another important distinction from the US [18, 29].

In larger municipalities, both income and racial SI were associated with lower odds of stillbirth, whereas the effects of both SI were weaker in smaller municipalities. Previous studies indicate that in larger cities, both income and racial segregation indices decrease in municipalities with a greater presence of precarious neighborhoods, such as favelas [18]. This occurs because favelas are typically multiracial, which lowers measured racial segregation, and they also reflect widespread poverty, which reduces measured income segregation at the municipal level. Consequently, low segregation scores in large cities often signal the presence of more favelas, and these municipalities tend to have higher odds of stillbirth. As for smaller municipalities, the Brazilian universal health coverage system, the Unified Health System (SUS), is often underfunded and understaffed in smaller cities [38, 39]. Thus, in smaller municipalities, the quality of SUS healthcare has a greater impact on the outcome than segregation.

This study has several limitations. First, the underreporting of stillbirths is possible, especially for the earlier weeks of gestation. We were unable to find previous reports on the magnitude of underreporting of stillbirths in SIM, which could lead to misclassification bias. Second, several critical unmeasured confounders were present. Factors such as family income and municipal SUS funding, which cannot be measured, are residual confounders. Also, studies from the US have shown that the effect of segregation is modified by maternal race [25, 32], but we failed to take this into account, as the SIM did not contain information about maternal race. Third, we could only measure segregation at the municipality level, and within-municipality segregation could not be considered. There are likely differences in segregation inside the municipality, especially in larger municipalities, and future studies using scales that are more sensitive to these within-municipality differences are needed. Lastly, we have yet to unveil the mechanisms by which income segregation affects birth outcomes in Brazilian society. Previous studies have proposed that hospital resource disparity [26] and neighborhood vulnerability to the withdrawal of resources distributed by the marketplace [40] are pathways through which segregation affects birth outcomes. More studies are needed to unveil these pathways and translate this finding into actionable policy plans.

## Conclusions

We have found that income segregation increases the risk of stillbirth, especially in regions where stillbirth incidence is relatively low. The association with racial segregation was less consistent. Brazil, like many Latin American countries, has a substantially segregated society, with stark income disparity; whereas races are more mixed and the racial segregation is less pronounced compared to the US. Our studies highlighted the harmful effects of income segregation and the heterogeneity of racial segregation in comparison to the US. Further studies with within-municipality segregation description and more information on the individual, such as race, are needed to investigate which people are affected by segregation and how.

## Supplementary Information

Supplementary Material 1.

## Data Availability

The data analyzed in this study were obtained from the publicly accessible DATASUS platform (http://www.datasus.gov.br). The datasets used and analyzed during the current study are available from the corresponding author upon reasonable request.

## Abbreviations

BMI: Body Mass Index
DATASUS: Departamento de Informações do Sistema Único de Saúde
DM: Diabetes Mellitus
IBGE: Instituto Brasileiro de Geografia e Estatística
IQR: Interquartile Range
LMIC: Low- or Middle-Income Countries
OR: Odds Ratio
PTB: Preterm Birth
SES: Socioeconomic Status
SI: Segregation Index
SIM: Sistema de Informações de Mortalidade
SINASC: Sistema de Informações de Nascidos Vivos
SUS: Sistema Único de Saúde
UCI: Upper Confidence Interval
