# Supplementary material for: The association between residential segregation and stillbirths in Brazil—a cross-sectional study
Source: BMC Pregnancy Childbirth. 2025 Nov 4;25:1162. doi: 10.1186/s12884-025-08329-x (PMC12584519; doi:10.1186/s12884-025-08329-x)
Supplement: Supplementary file 1 — Supplementary Material 1. [file 12884_2025_8329_MOESM1_ESM.pdf]

Supplementary material:

The Association Between Residential Segregation and Stillbirths in Brazil—A Cross-sectional Study

Authors:
Mio Kushibuchi1*, José Firmino Sousa Filho1, Andrêa J.F. Ferreira1, Hannah Blencowe2, Gervásio Ferreira Santos1, Mauricio L. Barreto1, Enny S. Paixão1-2.

Authors and Affiliations:
1 Centro de Integração de Dados e Conhecimentos para Saúde (CIDACS)
Centro de Integração de Dados e Conhecimentos de Saúde Edf Tecnocentro, Sl 315, R. Mundo, 121, Trobogy, Salvador, Bahia, Brazil
2 London School of Hygiene & Tropical Medicine (LSHTM)
Keppel Street, London, United Kingdom

Table of Contents

Supplementary Table 1: Details of the municipality-level variables for each income- and racial-SI quintiles..............3

Supplementary Table 2: Basic demographics by birth status (live birth or stillbirth)..4

Supplementary Table 3: Odds Ratio, 95% Confidence interval, and the p value for the OR of each SI quintile in the overall analyses, analyses stratified by city size, and for stillbirth type. 5

Supplementary Table 4: Odds Ratio, 95% Confidence interval, and the p value for the OR of each SI quintile in the overall analyses, analyses stratified by municipality stillbirth prevalence. 6

Supplementary Table 5: Descriptive statistics of stillbirth prevalence (mean, SD, minimum, and maximum) for area-level stillbirth prevalence strata. 7

Supplementary table 6: Municipality-level variable descriptions of the municipalities included in each strata. 9

Supplementary Table 7: Odds ratio for sensitivity analysis by region, excluding twins, and excluding the area poverty-level variable from the Logistic model..12

Supplementary Figure 1: Logical model for the association between segregation and stillbirth................................15

Supplementary Figure 2: The OR of income and racial SI quintiles for the analyses excluding twins and those lacking the variable...15

Supplementary Figure 3: The OR of income and racial SI quintiles for the analyses by regions. 16

Supplementary Figure 4: The OR of income and racial SI quintiles for overall population without the area poverty level variable in the logistic regression, and the same analyses done for tertile strata of area percentage of people earning less than half minimum wage. 17

Supplementary Table 1: Details of the municipality-level variables for each income- and racial-SI quintiles.

					Regions					Percentage of low-income people*		
	N municipality	median % under half SM	median population (thousand)	median stillbirth prevalence	Center-West	Northeast	North	Southeast	South	high (poor area)	middle	low (rich area)
Overall	5565	43.42 [25.33, 66.06]	10.9 [5.2, 23.4]	0.65 [0.00, 1.27]	1188 (21.3)	1794 (32.2)	466 (8.4)	1668 (30.0)	449 (8.1)	3108 (55.8)	1517 (27.3)	940 (16.9)
Income SI												
1st quintile	2617	31.02 [20.38, 49.59]	6.2 [3.7, 11.8]	0.00 [0.00, 1.12]	337 (12.9)	319 (12.2)	207 (7.9)	952 (36.4)	802 (30.6)	975 (37.3)	974 (37.2)	668 (25.5)
2nd quintile	1080	40.54 [24.74, 63.30]	14.4 [8.0, 30.4]	0.65 [0.00, 1.16]	86 (8.0)	291 (26.9)	93 (8.6)	398 (36.9)	212 (19.6)	578 (53.5)	318 (29.4)	184 (17.0)
3rd quintile	831	59.03 [34.54, 69.71]	17.7 [10.2, 34.7]	0.78 [0.32, 1.36]	34 (4.1)	390 (46.9)	73 (8.8)	200 (24.1)	134 (16.1)	586 (70.5)	178 (21.4)	67 (8.1)
4th quintile	644	67.50 [57.89, 73.38]	21.3 [12.6, 36.7]	0.96 [0.57, 1.55]	6 (0.9)	466 (72.4)	48 (7.5)	86 (13.4)	38 (5.9)	592 (91.9)	36 (5.6)	16 (2.5)
5th quintile	393	71.51 [65.25, 77.05]	20.0 [11.1, 36.1]	1.02 [0.58, 1.62]	3 (0.8)	328 (83.5)	28 (7.1)	32 (8.1)	2 (0.5)	377 (95.9)	11 (2.8)	5 (1.3)
Race SI												
1st quintile	1663	43.40 [28.05, 63.16]	7.8 [4.3, 16.8]	0.62 [0.00, 1.34]	231 (13.9)	459 (27.6)	179 (10.8)	625 (37.6)	169 (10.2)	952 (57.2)	521 (31.3)	190 (11.4)
2nd quintile	936	38.34 [24.06, 63.54]	14.4 [6.8, 31.9]	0.66 [0.00, 1.23]	86 (9.2)	295 (31.5)	64 (6.8)	337 (36.0)	154 (16.5)	472 (50.4)	305 (32.6)	159 (17.0)
3rd quintile	810	42.06 [23.97, 66.11]	15.1 [7.2, 31.9]	0.70 [0.00, 1.28]	67 (8.3)	283 (34.9)	45 (5.6)	276 (34.1)	139 (17.2)	444 (54.8)	201 (24.8)	165 (20.4)

4th quintile	709	45.30 [24.71, 66.98]	15.2 [7.3, 32.2]	0.70 [0.00, 1.21]	32 (4.5)	250 (35.3)	49 (6.9)	215 (30.3)	163 (23.0)	406 (57.3)	177 (25.0)	126 (17.8)
5th quintile	1447	48.17 [23.57, 70.18]	10.2 [4.7, 19.6]	0.58 [0.00, 1.26]	50 (3.5)	507 (35.0)	112 (7.7)	215 (14.9)	563 (38.9)	834 (57.6)	313 (21.6)	300 (20.7)

Footnotes: * Percentage of low income population was calculated by obtaining the perecentage of the municipality population earning fewer than half the minimum wage.

Supplementary Table 2: Basic demographics by birth status (live birth or stillbirth).

	Overall	Stillbirth	
		Live birth	Stillbirth
**Number**	2771272	2748512	22760
**Sex**			
Male	1419570 (51.2)	1407412 (51.2)	12158 (53.4)
Female	1351702 (48.8)	1341100 (48.8)	10602 (46.6)
**Maternal age**			
Median [IQR]	27.08 (6.67)	27.08 (6.67)	27.59 (7.28)
15 to 19 years	406010 (14.7)	402453 (14.6)	3557 (15.6)
20 to 34 years	1926156 (69.5)	1911638 (69.6)	14518 (63.8)
35 to 49 years	439106 (15.8)	434421 (15.8)	4685 (20.6)
**Maternal education**			
none to 3 years	62640 (2.3)	60569 (2.2)	2071 (9.1)
4 to 7 years	410100 (14.8)	404731 (14.7)	5369 (23.6)
8 to 11 years	1710744 (61.7)	1698535 (61.8)	12209 (53.6)
12 or more years	587788 (21.2)	584677 (21.3)	3111 (13.7)
**Gestation type**			
Singleton	2708059 (97.7)	2686730 (97.8)	21329 (93.7)
Twin or more	61424 (2.2)	60083 (2.2)	1341 (5.9)
missing	1789 (0.1)	1699 (0.1)	90 (0.4)
**Gestation weeks**			
<22 weeks	2790 (0.1)	1281 (0.0)	1509 (6.6)
22 to 27 weeks	18628 (0.7)	13458 (0.5)	5170 (22.7)
28 to 31 weeks	31292 (1.1)	27441 (1.0)	3851 (16.9)
32 to 36 weeks	265091 (9.6)	259210 (9.4)	5881 (25.8)
37 to 41 weeks	2352641 (84.9)	2347159 (85.4)	5482 (24.1)
42 or more weeks	69098 (2.5)	69001 (2.5)	97 (0.4)
missing	31732 (1.1)	30962 (1.1)	770 (3.4)

**Parity**			
Nulliparous	1735971 (62.6)	1719642 (62.6)	16329 (71.7)
Multiparous	1027396 (37.1)	1021136 (37.2)	6260 (27.5)
missing	7905 (0.3)	7734 (0.3)	171 (0.8)
**Previosu stillbirth**			
No history	2234364 (80.6)	2221444 (80.8)	12920 (56.8)
Stillbirth history	536908 (19.4)	527068 (19.2)	9840 (43.2)
**Municipality-level variables**			
**Region**			
Centro Oeste	240141 (8.7)	238224 (8.7)	1917 (8.4)
Nordeste	725320 (26.2)	717711 (26.1)	7609 (33.4)
Norte	284959 (10.3)	282307 (10.3)	2652 (11.7)
Sudeste	1128653 (40.7)	1120524 (40.8)	8129 (35.7)
Sul	392199 (14.2)	389746 (14.2)	2453 (10.8)
**Percent with low income***	34.72 (20.18)	34.69 (20.17)	38.84 (21.12)
**Municipality area**	2525.04 (8110.02)	2522.48 (8102.09)	2833.96 (9011.37)
**Municipality population (thousand)**	1201.6 (2786.2)	1202.5 (2787.8)	1090.7 (2589.6)

Footnotes: * Percentage of low income population was calculated by obtaining the perecentage of the municipality population earning fewer than half the minimum wage.

Supplementary Table 3: Odds Ratio, 95% Confidence interval, and the p value for the OR of each SI quintile in the overall analyses, analyses stratified by city size, and for stillbirth type.

	Income SI			Income SI		
	OR	95% CI	p value	OR	95% CI	p value
**Overall**						
1st quintile (least segregated)	Ref			Ref		
2nd quintile	0.976	0.935-1.019	0.266	0.932	0.894-0.971	0.001
3rd quintile	1.034	0.991-1.078	0.128	0.882	0.846-0.92	<0.001
4th quintile	0.886	0.848-0.926	<0.001	1.015	0.975-1.057	0.467
5th quintile (most segregated)	1.251	1.202-1.303	<0.001	0.945	0.908-0.984	0.006
**Stratification by city size below or above 500 thousand people**						
**Larger cities**						
1st quintile (least segregated)	Ref			Ref		
2nd quintile	0.793	0.676-0.931	0.004	0.753	0.676-0.839	<0.001
3rd quintile	0.756	0.652-0.876	<0.001	0.547	0.49-0.61	<0.001
4th quintile	0.388	0.337-0.447	<0.001	0.859	0.777-0.949	0.003

5th quintile (most segregated)	0.863	0.759-0.982	0.025	0.8	0.719-0.889	<0.001
**Smaller cities**						
1st quintile (least segregated)	Ref			Ref		
2nd quintile	0.975	0.932-1.019	0.263	0.933	0.891-0.976	0.003
3rd quintile	1.028	0.982-1.075	0.242	0.969	0.925-1.015	0.183
4th quintile	1.041	0.989-1.094	0.123	0.944	0.899-0.992	0.023
5th quintile (most segregated)	1.089	1.025-1.158	0.006	0.914	0.872-0.957	<0.001
**Analyses by stillbirth types**						
**Intrapartum stillbirths**						
1st quintile (least segregated)	Ref			Ref		
2nd quintile	0.986	0.838-1.161	0.868	0.908	0.777-1.061	0.226
3rd quintile	0.996	0.847-1.172	0.963	0.901	0.77-1.054	0.194
4th quintile	0.896	0.759-1.057	0.193	0.987	0.847-1.15	0.868
5th quintile (most segregated)	1.448	1.248-1.68	<0.001	1.065	0.92-1.233	0.398
**Antepartum stillbirth**						
1st quintile (least segregated)	Ref			Ref		
2nd quintile	0.975	0.933-1.019	0.257	0.934	0.895-0.974	0.001
3rd quintile	1.035	0.991-1.08	0.123	0.877	0.84-0.916	<0.001
4th quintile	0.886	0.848-0.927	<0.001	1.015	0.974-1.058	0.471
5th quintile (most segregated)	1.245	1.194-1.297	<0.001	0.931	0.894-0.971	0.001

Footnotes: The logistic model had the first quintile, the least segregad quintile, as the reference category. It was adjusted for maternal age, maternal education, previous stillbirth history, and area-level percentage of people earning less than half inimum wage. Abbreviations: LCI: lower confidence interval; OR: odds ratio; SI: segregation index; UCI: upper confidence interval.

Supplementary Table 4: Odds Ratio, 95% Confidence interval, and the p value for the OR of each SI quintile in the overall analyses, analyses stratified by municipality stillbirth prevalence.

	Income SI			Income SI		
	OR	95% CI	p value	OR	95% CI	p value
**Lowest**						
1st quintile (least segregated)	Ref			Ref		
2nd quintile	1.564	1.367-1.79	<0.001	1.102	0.945-1.286	0.216
3rd quintile	1.779	1.539-2.057	<0.001	1.153	0.982-1.355	0.083
4th quintile	1.744	1.449-2.1	<0.001	1.117	0.934-1.335	0.224
5th quintile (most segregated)	2.086	1.494-2.911	<0.001	0.923	0.783-1.089	0.345

**Second lowest**						
1st quintile (least segregated)	Ref			Ref		
2nd quintile	0.991	0.875-1.122	0.885	1.061	0.93-1.21	0.379
3rd quintile	0.995	0.879-1.126	0.936	1.039	0.924-1.168	0.525
4th quintile	0.48	0.42-0.549	<0.001	0.529	0.463-0.604	<0.001
5th quintile (most segregated)	1.004	0.883-1.141	0.955	0.989	0.858-1.14	0.879
**Third lowest**						
1st quintile (least segregated)	Ref			Ref		
2nd quintile	1.033	0.917-1.165	0.594	1.015	0.909-1.134	0.786
3rd quintile	1.012	0.896-1.143	0.849	0.983	0.876-1.104	0.777
4th quintile	0.913	0.814-1.024	0.119	1.544	1.396-1.709	<0.001
5th quintile (most segregated)	1.419	1.289-1.563	<0.001	0.95	0.854-1.055	0.338
**Fourth lowest**	Ref			Ref		
1st quintile (least segregated)						
2nd quintile	1.062	0.969-1.165	0.197	1.031	0.954-1.115	0.438
3rd quintile	1.079	0.994-1.172	0.069	0.763	0.695-0.837	<0.001
4th quintile	1.095	0.996-1.205	0.061	1.325	1.217-1.442	<0.001
5th quintile (most segregated)	1.038	0.945-1.141	0.433	1.035	0.948-1.13	0.445
**Highest prevalence**						
1st quintile (least segregated)	Ref			Ref		
2nd quintile	0.874	0.815-0.936	<0.001	0.943	0.881-1.009	0.09
3rd quintile	0.876	0.816-0.941	<0.001	0.991	0.925-1.062	0.807
4th quintile	0.849	0.792-0.91	<0.001	0.939	0.879-1.004	0.065
5th quintile (most segregated)	0.846	0.792-0.904	<0.001	0.952	0.896-1.01	0.105

Footnotes: The logistic model had the first quintile, the least segregated quintile, as the reference category. It was adjusted for maternal age, maternal education, previous stillbirth history, and area-level percentage of people earning less than half minimum wage. Abbreviations: LCI: lower confidence interval; OR: odds ratio; SI: segregation index; UCI: upper confidence interval.

Supplementary Table 5: Descriptive statistics of stillbirth prevalence (mean, SD, minimum, and maximum) for area-level stillbirth prevalence strata.

	Number of stillbirths per 1000 birth

Stillbirth prevalence strata	total number of births	Mean	SD	Minimum	Maximum
least prevalent	600,245	4.58	2.51	0.00	7.09
2nd least	581,427	8.19	0.63	7.11	9.20
3rd least	617,606	9.63	0.30	9.20	10.13
4th least	572,977	11.40	0.81	10.14	12.89
most prevalent	580,984	17.63	5.73	12.90	100.00

SD: standard deviation

Supplementary table 6: Municipality-level variable descriptions of the municipalities included in each strata.

						Regions					percentage of low-income people*			quintile of income SI					Quintile of racial SI				
	N municipality	N participants	Median percentage with low-income	Median population (thousand)	Median stillbirth prevalence	Center-west	Northeast	North	Southeast	South	high	middle	low	1st	2nd	3rd	4th	5th	1st	2nd	3rd	4th	5th
**Stratification by municpality population size**																							
Large city	38	779105	20.74 [15.56, 34.81]	809.0 [611.0, 1405.3]	0.78 [0.65, 0.93]	4 (10.5)	11 (28.9)	2 (5.3)	17 (44.7)	4 (10.5)	4 (10.5)	15 (39.5)	19 (50.0)	2 (5.3)	5 (13.2)	7 (18.4)	10 (26.3)	14 (36.8)	4 (10.5)	10 (26.3)	10 (26.3)	7 (18.4)	7 (18.4)
small city	5527	1992167	43.85 [25.44, 66.12]	108.7 [5.2, 2.2]	0.64 [0.00, 1.28]	462 (8.4)	1783 (32.3)	447 (8.1)	1651 (29.9)	1184 (21.4)	3104 (56.2)	1502 (27.2)	921 (16.7)	2615 (47.3)	1075 (19.4)	824 (14.9)	634 (11.5)	379 (6.9)	1659 (30.0)	926 (16.8)	800 (14.5)	702 (12.7)	1440 (26.1)
**Staratification by area stillbirth prevalence**																							
lowest	2511	554254	34.75 [21.99, 58.73]	6.5 [3.7, 14.8]	0.00 [0.00, 0.00]	201 (8.0)	531 (21.1)	147 (5.9)	895 (35.6)	737 (29.4)	1142 (45.5)	803 (32.0)	566 (22.5)	1526 (60.8)	459 (18.3)	284 (11.3)	149 (5.9)	93 (3.7)	788 (31.4)	414 (16.5)	324 (12.9)	281 (11.2)	704 (28.0)
2nd lowest	372	554254	35.60 [21.01, 65.50]	24.7 [14.1, 58.1]	0.62 [0.58, 0.65]	41 (11.0)	102 (27.4)	41 (11.0)	103 (27.7)	85 (22.8)	175 (47.0)	107 (28.8)	90 (24.2)	99 (26.6)	112 (30.1)	76 (20.4)	59 (15.9)	26 (7.0)	82 (22.0)	68 (18.3)	71 (19.1)	67 (18.0)	84 (22.6)

3rd lowest	394	554254	39.62 [22.95, 66.14]	25.7 [13.7, 61.1]	0.75 [0.71, 0.78]	34 (8.6)	138 (35.0)	40 (10.2)	113 (28.7)	69 (17.5)	203 (51.5)	111 (28.2)	80 (20.3)	121 (30.7)	86 (21.8)	82 (20.8)	63 (16.0)	42 (10.7)	96 (24.4)	75 (19.0)	63 (16.0)	69 (17.5)	91 (23.1)
4th lowest	545	554254	48.57 [27.12, 67.83]	22.9 [11.4, 48.8]	0.94 [0.88, 1.01]	55 (10.1)	208 (38.2)	55 (10.1)	144 (26.4)	83 (15.2)	321 (58.9)	152 (27.9)	72 (13.2)	181 (33.2)	118 (21.7)	106 (19.4)	92 (16.9)	48 (8.8)	142 (26.1)	101 (18.5)	83 (15.2)	84 (15.4)	135 (24.8)
most	1748	554254	59.57 [35.53, 70.14]	11.0 [5.9, 21.3]	1.68 [1.32, 2.38]	136 (7.8)	816 (46.7)	167 (9.6)	414 (23.7)	215 (12.3)	1268 (72.5)	345 (19.7)	135 (7.7)	691 (39.5)	306 (17.5)	284 (16.2)	283 (16.2)	184 (10.5)	555 (31.8)	279 (16.0)	270 (15.4)	209 (12.0)	435 (24.9)
**Stratification by region**																							
North	449	284815	64.19 [53.60, 74.46]	14.9 [6.6, 30.0]	0.81 [0.26, 1.39]						425 (94.7)	24 (5.3)	0	207 (46.1)	93 (20.7)	73 (16.3)	48 (10.7)	28 (6.2)	179 (39.9)	64 (14.3)	45 (10.0)	49 (10.9)	112 (24.9)
Norhteast	1794	724970	68.66 [62.80, 73.64]	13.8 [7.3, 24.6]	0.98 [0.37, 1.66]						1778 (99.1)	15 (0.8)	1 (0.1)	319 (17.8)	291 (16.2)	390 (21.7)	466 (26.0)	328 (18.3)	459 (25.6)	295 (16.4)	283 (15.8)	250 (13.9)	507 (28.3)
South	1188	392087	23.42 [14.64, 33.24]	7.0 [3.7, 16.3]	0.00 [0.00, 0.82]						190 (16.0)	498 (41.9)	500 (42.1)	802 (67.5)	212 (17.8)	134 (11.3)	38 (3.2)	2 (0.2)	169 (14.2)	154 (13.0)	139 (11.7)	163 (13.7)	563 (47.4)
Southeast	1668	1128304	29.67 [20.92, 43.91]	10.6 [5.2, 25.8]	0.45 [0.00, 1.07]						552 (33.1)	711 (42.6)	405 (24.3)	952 (57.1)	398 (23.9)	200 (12.0)	86 (5.2)	32 (1.9)	625 (37.5)	337 (20.2)	276 (16.5)	215 (12.9)	215 (12.9)

Center-west	466	240069	33.24 [27.05, 41.35]	8.5 [4.1, 19.0]	0.66 [0.00, 1.17]						163 (35.0)	269 (57.7)	34 (7.3)	337 (72.3)	86 (18.5)	34 (7.3)	6 (1.3)	3 (0.6)	231 (49.6)	86 (18.5)	67 (14.4)	32 (6.9)	50 (10.7)
**Stratification by the percentage of people earning lower than half the minimum wage***																							
High percentage	3109	902366	64.32 [52.71, 71.71]	11.2 [5.7, 21.4]	0.85 [0.00, 1.54]	163 (5.2)	1779 (57.2)	425 (13.7)	552 (17.8)	190 (6.1)				975 (31.4)	578 (18.6)	586 (18.8)	592 (19.0)	378 (12.2)	952 (30.6)	473 (15.2)	444 (14.3)	406 (13.1)	834 (26.8)
Middle percentage	1518	902367	28.60 [24.67, 32.92]	10.3 [4.6, 24.1]	0.46 [0.00, 1.01]	269 (17.7)	15 (1.0)	24 (1.6)	712 (46.9)	498 (32.8)				974 (64.2)	318 (20.9)	178 (11.7)	36 (2.4)	12 (0.8)	521 (34.3)	305 (20.1)	201 (13.2)	177 (11.7)	314 (20.7)
Low percentage	940	902367	15.18 [11.23, 17.91]	11.8 [4.7, 39.4]	0.37 [0.00, 0.76]	34 (3.6)	1 (0.1)	0	405 (43.1)	500 (53.2)				668 (71.1)	184 (19.6)	67 (7.1)	16 (1.7)	5 (0.5)	190 (20.2)	159 (16.9)	165 (17.6)	126 (13.4)	300 (31.9)

Footnotes: * Percentage of low income population was calculated by obtaining the perecentage of the municipality population earning fewer than half the minimum wage.

Supplementary Table 7: Odds ratio for sensitivity analysis by region, excluding twins, and excluding the area poverty-level variable from the Logistic model.

	Income SI			Income SI		
	OR	95% CI	p value	OR	95% CI	p value
**Region**						
**North region**						
1st quintile (least segregated)	Ref			Ref		
2nd quintile	1.077	0.94-1.235	0.285	0.93	0.823-1.05	0.239
3rd quintile	1.202	1.045-1.384	0.01	0.502	0.43-0.585	<0.001
4th quintile	1.096	0.962-1.249	0.169	2.111	1.874-2.378	<0.001
5th quintile (most segregated)	1.186	1.019-1.38	0.028	1.43	1.242-1.646	<0.001
**Northeast region**						
1st quintile (least segregated)	Ref			Ref		
2nd quintile	1.041	0.926-1.169	0.501	0.914	0.847-0.987	0.022
3rd quintile	1.055	0.947-1.175	0.331	0.919	0.851-0.992	0.03
4th quintile	1.101	0.996-1.216	0.06	0.996	0.923-1.075	0.919
5th quintile (most segregated)	1.11	1.005-1.225	0.039	1.005	0.933-1.082	0.899
**Southeast region**						
1st quintile (least segregated)	Ref			Ref		
2nd quintile	0.911	0.852-0.974	0.006	0.891	0.831-0.955	0.001
3rd quintile	0.984	0.919-1.054	0.643	0.823	0.761-0.89	<0.001
4th quintile	0.489	0.447-0.534	<0.001	0.938	0.879-1.001	0.053
5th quintile (most segregated)	1.575	1.476-1.681	<0.001	0.948	0.884-1.016	0.133
**South region**						
1st quintile (least segregated)	Ref			Ref		
2nd quintile	1.038	0.931-1.157	0.505	1.214	1.017-1.449	0.032
3rd quintile	1.09	0.983-1.21	0.102	1.155	0.966-1.381	0.114
4th quintile	1.062	0.93-1.212	0.373	1.1	0.918-1.317	0.301
5th quintile (most segregated)	0.713	0.099-5.142	0.737	1.057	0.891-1.253	0.525
**Center west region**						
1st quintile (least segregated)	Ref			Ref		
2nd quintile	1.041	0.914-1.186	0.546	0.98	0.856-1.122	0.769
3rd quintile	1.113	0.978-1.266	0.106	0.902	0.793-1.025	0.114
4th quintile	1.12	0.901-1.392	0.308	1.149	0.908-1.453	0.248

5th quintile (most segregated)	0.848	0.722-0.996	0.044	0.926	0.733-1.17	0.519
**Sensitivity analyses excluding twins**						
1st quintile (least segregated)	Ref			Ref		
2nd quintile	0.98	0.936-1.025	0.375	0.927	0.888-0.968	0.001
3rd quintile	1.033	0.988-1.08	0.154	0.865	0.828-0.905	<0.001
4th quintile	0.884	0.844-0.926	<0.001	1.002	0.961-1.046	0.914
5th quintile (most segregated)	1.246	1.193-1.3	<0.001	0.921	0.883-0.961	<0.001
**Sensitivity analyses excluding area-level poverty variable from the logistic model**						
**Overall**						
1st quintile (least segregated)	Ref			Ref		
2nd quintile	0.982	0.939-1.028	0.441	0.913	0.874-0.954	<0.001
3rd quintile	1.053	1.007-1.101	0.023	0.846	0.81-0.885	<0.001
4th quintile	0.933	0.892-0.977	0.003	0.985	0.944-1.028	0.488
5th quintile (most segregated)	1.287	1.234-1.343	<0.001	0.929	0.89-0.969	0.001
**Strata with highest percentage of people with low income**						
1st quintile (least segregated)	Ref			Ref		
2nd quintile	1.037	0.956-1.125	0.383	0.937	0.876-1.003	0.061
3rd quintile	1.084	1.003-1.172	0.041	0.987	0.925-1.054	0.707
4th quintile	1.116	1.039-1.199	0.003	0.977	0.91-1.049	0.517
5th quintile (most segregated)	1.098	1.017-1.185	0.016	0.977	0.918-1.039	0.456
**Strata with middle percentage of people with low income**						
1st quintile (least segregated)	Ref			Ref		
2nd quintile	0.984	0.917-1.056	0.652	0.938	0.879-1.001	0.053
3rd quintile	0.978	0.911-1.05	0.54	0.627	0.575-0.684	<0.001
4th quintile	0.985	0.899-1.08	0.754	1.178	1.099-1.263	<0.001
5th quintile (most segregated)	1.261	1.181-1.347	<0.001	1.118	1.04-1.201	0.002
**Strata with lowest percentage of people with low income**						
1st quintile (least segregated)	Ref			Ref		
2nd quintile	0.965	0.882-1.056	0.441	1.091	0.947-1.255	0.227
3rd quintile	1.151	1.053-1.259	0.002	1.161	1.016-1.328	0.028
4th quintile	0.521	0.468-0.581	<0.001	1.229	1.077-1.401	0.002
5th quintile (most segregated)	1.644	1.514-1.786	<0.001	0.999	0.871-1.146	0.988

Footnotes: The logistic model had the first quintile, the least segregated quintile, as the reference category. For the region-stratified and the analyses excluding twins, it was adjusted for maternal age, maternal education, previous stillbirth history, and area-level percentage of people earning less than half minimum wage. For the analyses excluding the area poverty level variable (overall and by area poverty tertile), the analyses

was adjusted for maternal age, maternal education, and previous stillbirth history. Abbreviations: LCI: lower confidence interval; OR: odds ratio; SI: segregation index; UCI: upper confidence interval.

Supplementary Figure 1: Logical model for the association between segregation and stillbirth.

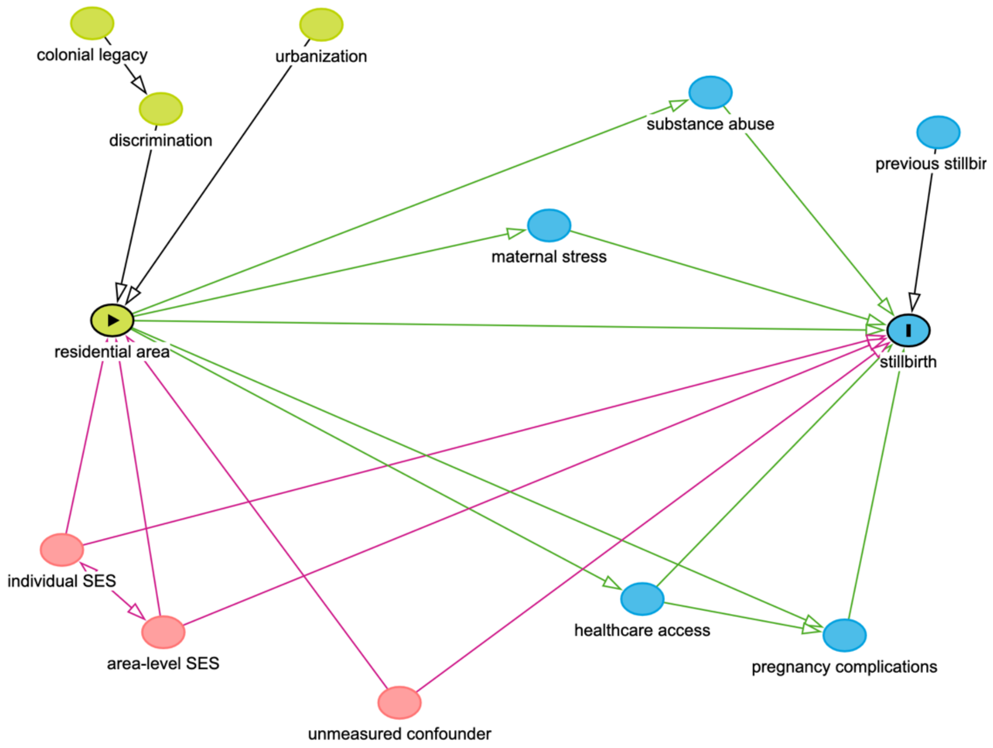

Supplementary Figure 2: The OR of income and racial SI quintiles for the analyses excluding twins and those lacking the variable.

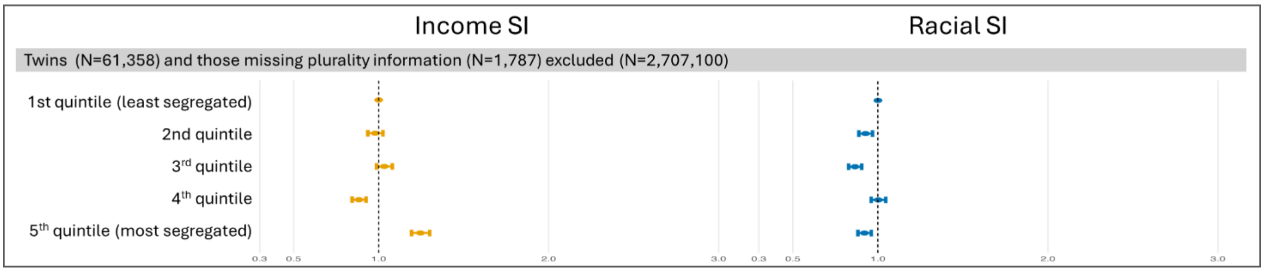

All the OR are adjusted for maternal age, maternal education, child sex, previous stillbirth, and municipality-level percentage earning less than half minimum wage. Abbreviations: OR, odds ratio; SI, segregation index.

Supplementary Figure 3: The OR of income and racial SI quintiles for the analyses by regions.

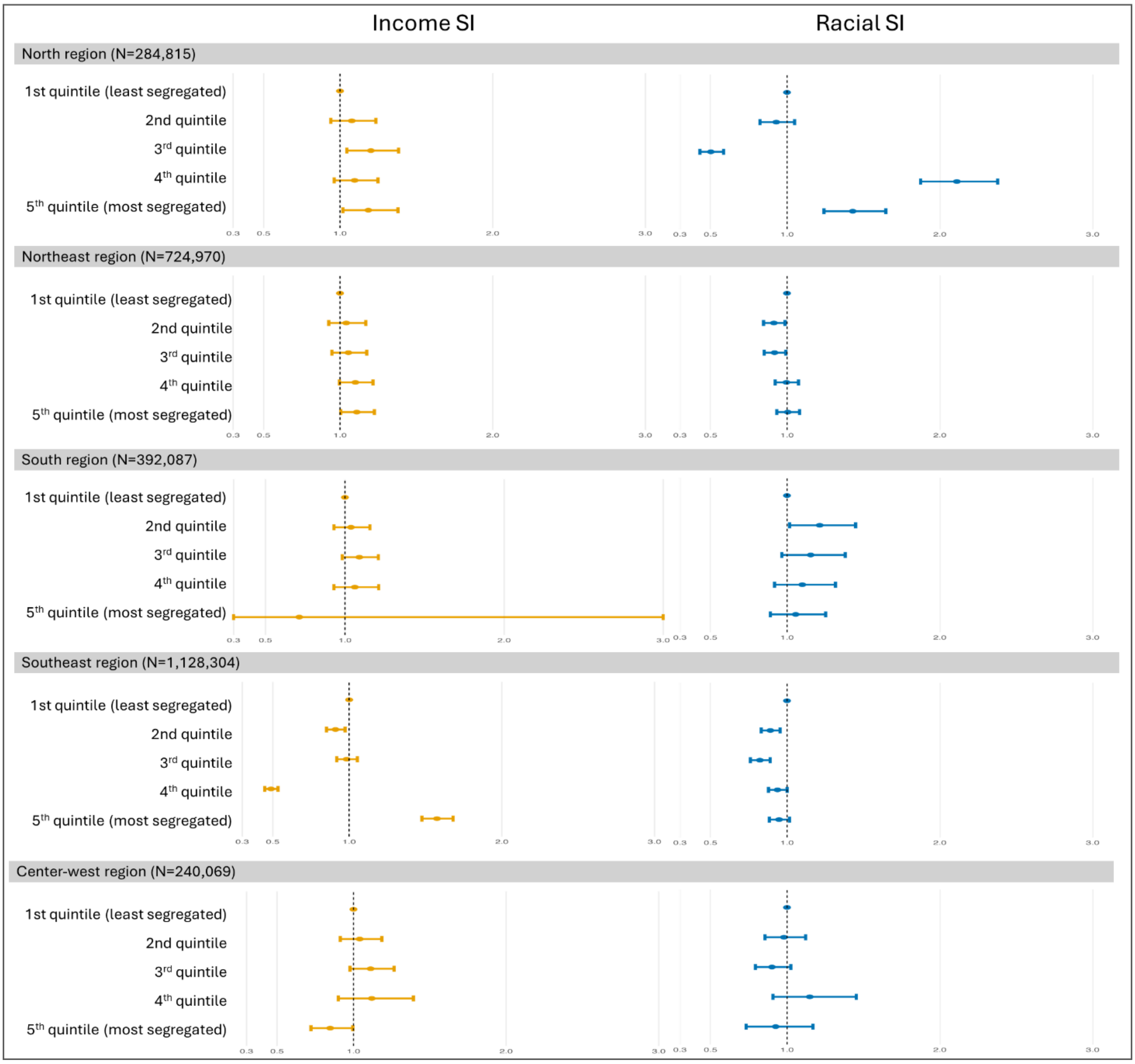

All the OR are adjusted for maternal age, maternal education, child sex, previous stillbirth, and municipality-level percentage earning less than half minimum wage. Abbreviations: OR, odds ratio; SI, segregation index.

Supplementary Figure 4: The OR of income and racial SI quintiles for overall population without the area poverty level variable in the logistic regression, and the same analyses done for tertile strata of area percentage of people earning less than half minimum wage.

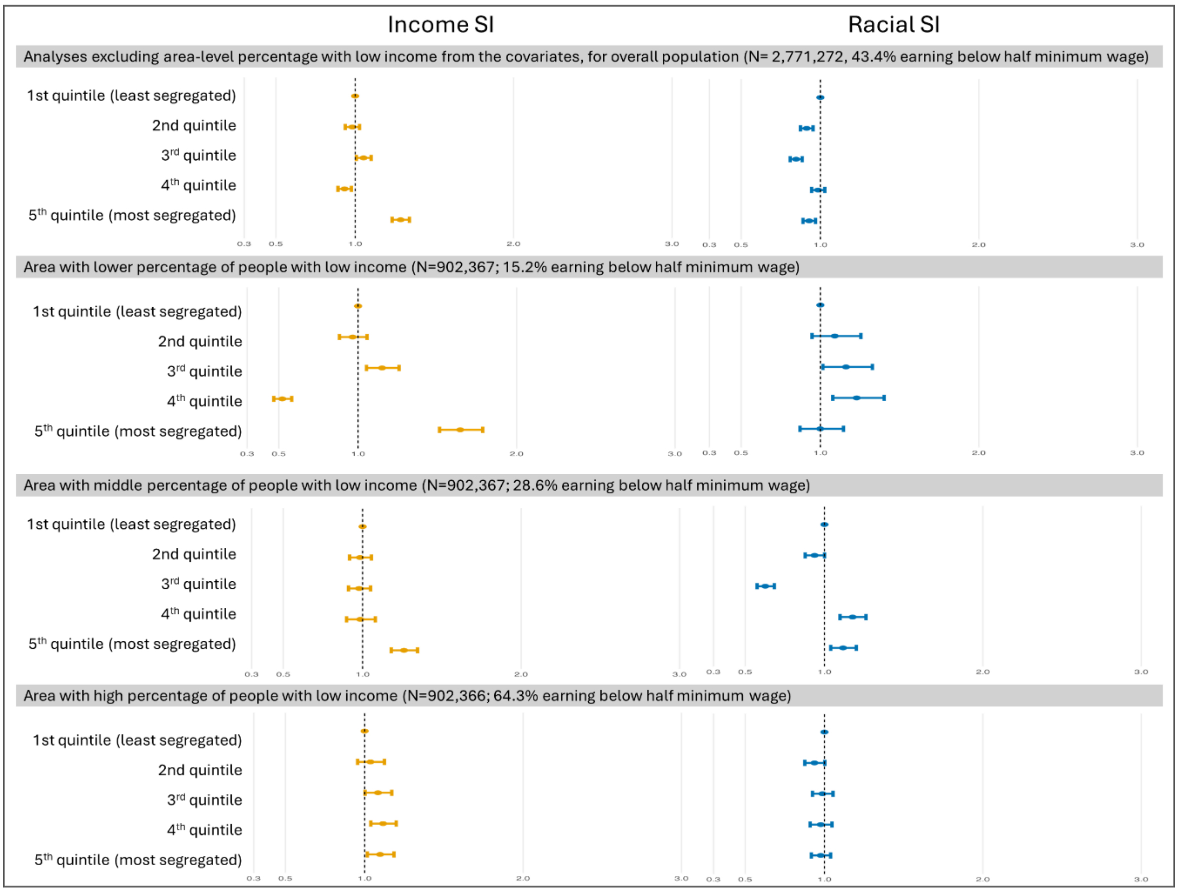

All the OR are adjusted for maternal age, maternal education, child sex, and previous stillbirth. Abbreviations: OR, odds ratio; SI, segregation index.
